# Anatomy of Mitral Valve Complex as Revealed by Non-Invasive Imaging: Pathological, Surgical and Interventional Implications

**DOI:** 10.3390/jcdd7040049

**Published:** 2020-11-04

**Authors:** Laura Anna Leo, Vera Lucia Paiocchi, Susanne Anna Schlossbauer, Elisa Gherbesi, Francesco F. Faletra

**Affiliations:** Department of Cardiology, Fondazione Cardiocentro Ticino, Via Tesserete 48, CH-6900 Lugano, Switzerland; lauraanna.leo@cardiocentro.org (L.A.L.); vera.paiocchi@cardiocentro.org (V.L.P.); susanne.schlossabauer@cardiocentro.org (S.A.S.); elisa.gherbesi@cardiocentro.org (E.G.)

**Keywords:** mitral valve anatomy, multimodality imaging, two-dimensional and three-dimensional transthoracic and transesophageal echocardiography, computed tomography, cardiac magnetic resonance

## Abstract

Knowledge of mitral valve (MV) anatomy has been accrued from anatomic specimens derived by cadavers, or from direct inspection during open heart surgery. However, today two-dimensional and three-dimensional transthoracic (2D/3D TTE) and transesophageal echocardiography (2D/3D TEE), computed tomography (CT) and cardiac magnetic resonance (CMR) provide images of the beating heart of unprecedented quality in both two and three-dimensional format. Indeed, over the last few years these non-invasive imaging techniques have been used for describing dynamic cardiac anatomy. Differently from the “dead” anatomy of anatomic specimens and the “static” anatomy observed during surgery, they have the unique ability of showing “dynamic” images from beating hearts. The “dynamic” anatomy gives us a better awareness, as any single anatomic arrangement corresponds perfectly to a specific function. Understanding normal anatomical aspects of MV apparatus is of a paramount importance for a correct interpretation of the wide spectrum of patho-morphological MV diseases. This review illustrates the anatomy of MV as revealed by non-invasive imaging describing physiological, pathological, surgical and interventional implications related to specific anatomical features of the MV complex.

## 1. Introduction

**Figure 1:**
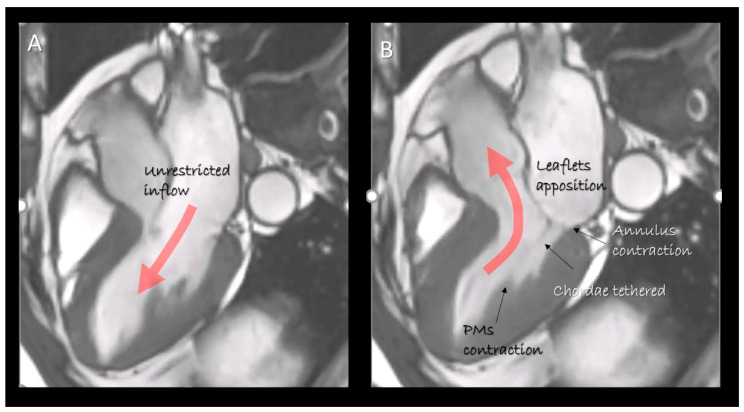
Cardiac magnetic resonance (CMR) still frame image in cross-section long-axis view in (**A**) diastole and (**B**) systole showing the mitral valve apparatus formed by leaflets, annulus, chordae tendinea and papillary muscles. They move in a perfect spatial and temporal coordination to obtain an effective competence and an unrestricted inflow. The “dynamic” anatomy provided by non-invasive imaging techniques gives us the awareness as the structural arrangement of any single component serves to a specific function.

Traditionally the knowledge of mitral valve (MV) anatomy has been accrued from anatomic specimens derived by cadavers, or from a direct inspection during open heart surgery. Currently, two-dimensional and three-dimensional transthoracic (2D/3D TTE) and transesophageal echocardiography (2D/3D TEE), computed tomography (CT) and cardiac magnetic resonance (CMR) provide images of the beating heart of unprecedented quality in both two and three-dimensional format. Indeed, over the last few years these non-invasive imaging techniques have been used for describing cardiac anatomy [1,2,3,4,5,6,7,8,9]. Differently from the “dead” anatomy of anatomic specimens and the “static” anatomy observed during surgery, these techniques have the unique ability to show “dynamic” images from beating hearts. The “dynamic” anatomy is a novel concept that gives us a better understanding as any single anatomic structure corresponds perfectly to a specific function. This is particularly true for the MV apparatus (Figure 1).

As emphasized by Perloff and Williams in their seminal paper [10], MV is not a mere couple of tissue flaps which open and close, following pressure fluctuations between the left atrium (LA) and the left ventricle (LV). On the contrary, the valve is a much more complex apparatus in which several components move in a perfect spatial and temporal coordination to ensure an effective valve competence and an unrestricted inflow. Key components of this dynamic three-dimensional apparatus are mitral annulus, leaflets, chordae tendineae and papillary muscles (PMs). 

This review describes the anatomy of the components of the MV apparatus, as revealed by the aforementioned non-invasive imaging techniques, highlighting physiological, pathological, surgical and interventional implications related to specific anatomical structures (Figure 1).

## 2. Mitral Annulus

The notion that the mitral annulus (MA) is a ring of connective tissue that encircles the left atrioventricular junction and anchors the leaflets is rather misleading. The anatomical arrangement *“left atrial wall-fibrous circular ring-left ventricular wall”* does not exist. Indeed, instead of a circular ring, anatomists described the MA as consisting of a straight anterior and a C-shaped posterior segment, completely different in their structure and function [11]. 

**Figure 2:**
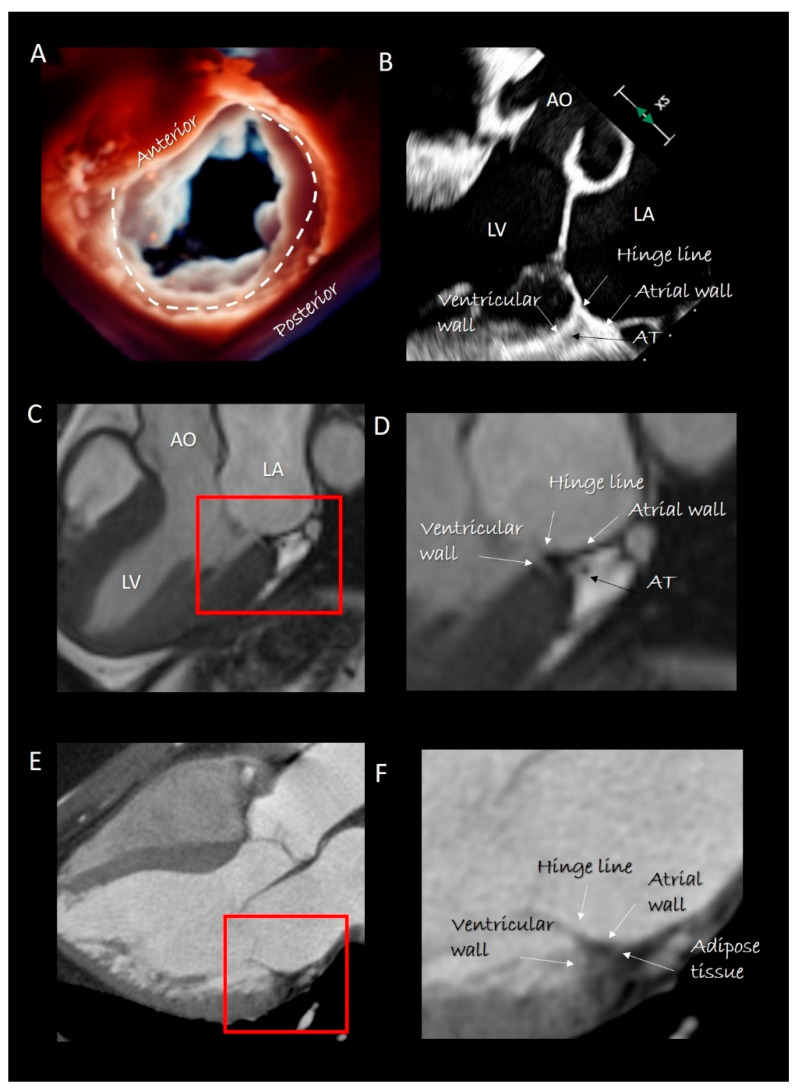
(**A**) 3D transesophageal echocardiography (TEE) still frame image of mitral valve forms an overhead perspective with a specific rendering algorithm that permits a movable source of light. With a source of light behind the valve, the two components of mitral annulus are well visible (the curved dotted line marks the posterior segment). (**B**) 2D TEE cross-section long-axis view showing the four components of posterior segment of annulus. The adipose tissue (AT) has a different texture compared with surrounding tissues. (**C**) CMR cross-section long-axis view. (**D**) Magnified image of the structures inside the red square in **C** showing the four components of posterior segment. The AT can be distinguished because the signal is much stronger (white in color) than that of surrounding structures. (**E**) Computed tomography (CT) cross-section long-axis view. (**F**) Magnified image of the structures inside the red square in **E** showing the four components of the posterior segment. As AT is more hypodense to x-ray, it appears as an area that is darker than the surrounding structures. AO = aorta; LV = left ventricle; LA = left atrium.

***The posterior segment*** anchors the posterior leaflet and can be roughly depicted as a “C-shaped” structure covering the posterior aspect of the MA from the left to the right trigones. This segment is actually the result of the convergence of four structures: the atrial wall, the leaflet hinge line, the marginal free wall of left ventricle and adipose tissue (AT) (Figure 2). A thin fibrous string (which is actually the “true” posterior annulus) “glues” these components together. However, this fibrous string is discontinuous and in those parts in which it is absent, the posterior leaflet is inserted directly on the junction of ventricular and atrial myocardium [12]. The sphincteric-like contraction of the MA is facilitated by this anatomical arrangement. Indeed, those parts of the posterior hinge line directly attached on ventricular myocardium follow the contraction of LV reducing the MA area by 20–30% [13]. The smallest area occurs in isovolumetric contraction preparing an effective leaflet coaptation, while the largest area of MA is seen in the isovolumetric relaxation, anticipating the torrential early filling. Notably, in this “pre-systolic” phase, atrial contraction plays a fundamental role, confirming that the atrial myocardium is an integrant part of posterior MA [14].

***The anterior segment*** of the MA is actually made up only by the hinge line of the anterior mitral leaflet (AML). In its ventricular aspect, this hinge line continues with the mitral–aortic curtain, a strip of fibrous tissue delimited medially and laterally by two robust fibrous nodules called trigones. The mitral aortic curtain gradually merges with the left interleaflet triangle (ILT) located between the non-coronary and left coronary sinus. However, there is marked normal variation in this relationship depending on the normal variation seen in the rotational position of the aortic root [15]. Since there are no anatomical boundaries between the ventricular surface of the AML, the mitral–aortic curtain and the aortic ILT, these components should be considered as a single structure. From an atrial perspective the hinge line of the AML is in direct continuity with the atrial wall.

***The saddle-shaped MA:*** Challenging the common belief that the MA is planar, Levine et al. in 1987 demonstrated that the MA actually has a three-dimensional aspect similar to a saddle, with “peaks” being located at the level of the midpoints of the anterior and posterior leaflets and “valleys” at the commissural level [16]. Using computerized finite elements analysis, Salgo et al. [17] demonstrated that this anatomical arrangement confers a mechanical advantage reducing stress on mitral leaflets by increasing their curvature. Since this saddle-shaped configuration is preserved across mammalian species, it can be speculated that the “saddle annulus” has gradually evolved through millions of years to preserve the integrity of leaflets, likely as the result of minute changes caused by application of varying degree of forces all along the annular circumference. However, saying that the mitral annulus has a saddle-shaped aspect is anatomically incorrect. Indeed, neither in Robert Levine’s original paper, nor in the present-day, using dedicated software, the contour to reconstruct the MA is drawn on the anatomical annulus but rather on the hinge line of leaflets. Indeed, when present, the anatomical annulus is located 2–3 mm external to the hinge line. Thus, the saddle-shaped aspect refers to the hinge line of leaflets, rather than to the actual anatomical annulus.

### Imaging Techniques

**Figure 3:**
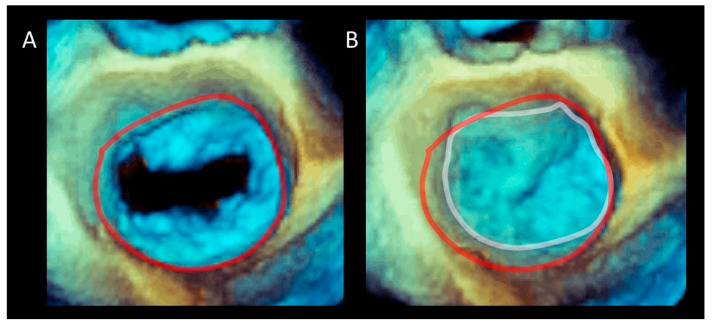
Three-dimensional TEE image of the mitral valve (MV) from an overhead perspective in (**A**) diastole and (**B**) systole. The sphincteric action of the annulus is marked with a red (diastolic) and white (systolic) circumference.

**Figure 4:**
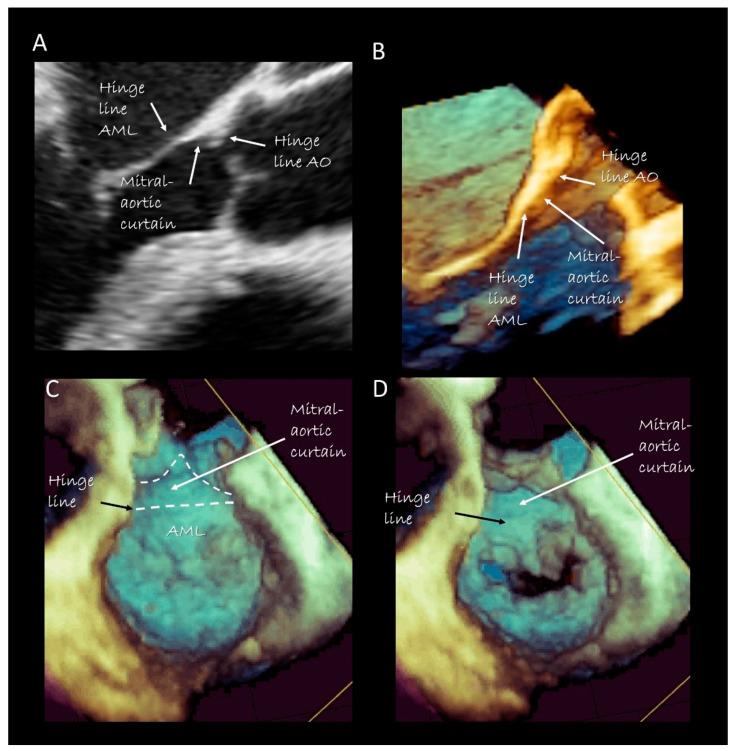
Two-dimensional (**A**) and three-dimensional (**B**) TEE cross-section long-axis view showing the hinge line of anterior mitral leaflet (AML), the hinge line of the aorta (AO) and in between the mitral aortic curtain. (**C**,**D**) 3D TEE from a ventricular perspective in systole (**C**) and in diastole (**D**), showing the mitral–aortic curtain in “en face” view.

Cross sections obtained by 2D TTE and TEE, CMR and CT perfectly portray these anatomical details (Figure 2). Three-dimensional TEE from an overhead perspective shows unique images of the entire hinge line of leaflets. Notably, in the cross section, the fibrous posterior annulus should appear as a fibrous nodule. It must be emphasized that none of the above-mentioned techniques is capable of visualizing this fibrous nodule as a distinct structure. Thus, the only means to visualize the posterior annulus is through histological specimens. The absence of a rigid “C-shaped” semi-annulus facilitates the sphincteric-like contraction. Those segments of posterior leaflet attached directly on ventricular myocardium freely follow contraction and relaxation of left ventricle (Figure 3). CMR shows that from the atrioventricular groove, the adipose tissue (AT) penetrates deeply up to the base of posterior leaflet. This “intrusiveness”, along with the fibrous skeleton of the heart, may contribute to the electrical insulation between the atrial and ventricular musculature and may provide a mechanical protection to coronary artery buffering and to the torsion induced by arterial pulse and myocardial contraction. Cross-sectional planes 2D/3D TEE, CT scan and CMR show as the hinge line of the anterior leaflet is more apically displaced than the hinge line of aortic leaflet. The space between the two hinge lines is occupied by the above-mentioned mitral–aortic curtain (Figure 4A,B). Three-dimensional TEE permits visualizing the mitral–aortic curtain from an “en face” perspective showing as the base of the AML, its hinge line, the mitral–aortic curtain and the ILT, which appear as a single area with no boundaries (Figure 4C,D).

Quantification of annular size can be best obtained by CT and 3D TEE. Being intrinsically three-dimensional, both techniques, in fact, provide true volumetric images of the mitral annulus. CT sizing (obtained either using specialized software or tracing manually the MA contour) is more accurate and reproducible than 3D TEE, due to its higher spatial isotropic resolution [18]. Currently, with the advent of transcatheter mitral valve replacement, CT has become the method of choice in the assessment of MA dimension and for predicting LV outflow obstruction after transcutaneous valve replacement [19]. However, it must be said that CT has quite a few limitations: first, the “dynamic” annular sizing (i.e., measurements in systole and diastole) requires coverage of the entire cardiac cycle with no dose modulation. With this approach, even with the last generation of CT scanner, the radiation dose is significantly higher than routine CT scanning increasing the risk of cancer-related irradiation [20]. Second, the temporal resolution remains inferior to the 3D TEE (up to 20 Hz, but usually 10 Hz vs. 50 Hz) and third, iodinate contrast material may cause additional renal injury in patients with renal impairment. 

## 3. Mitral Leaflets

**Figure 5:**
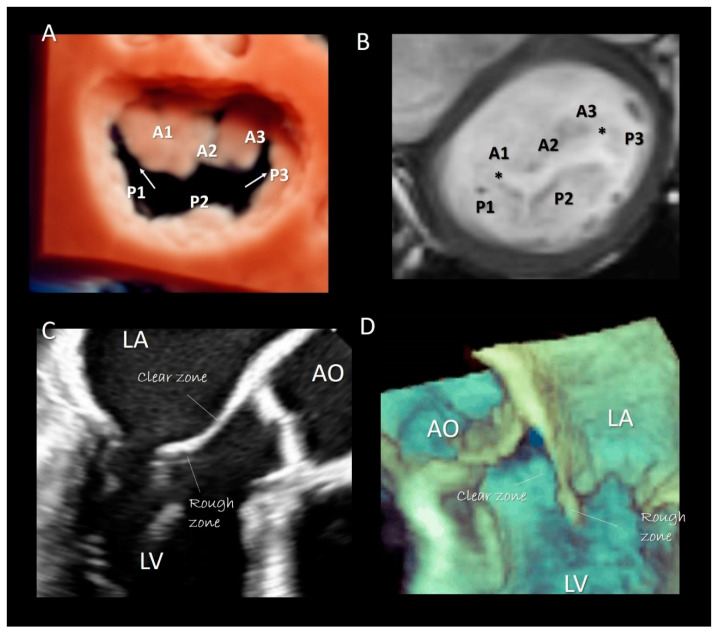
(**A**) 3D TEE with a new volume rendering algorithm from an overhead view, showing the two commissure (arrows) and the P1, P2, P3 scallops of the posterior leaflet. A1, A2, A3 are the corresponding segments of anterior leaflet. (**B**) CMR cross-section short-axis at the level of mitral leaflets showing the commissures (asterisks) and the scallops. (**C**) 2D TEE and (**D**) 3D TEE cross-section long-axis views showing the rough and clear zones.

Two deep commissures separate the mitral veil in two halves. The anterior (or aortic) mitral leaflet (AML) and the posterior (or mural) mitral leaflet (PML). At both ends of the zones of apposition between the aortic and mural leaflets can often be found commissural leaflets, which occasionally can be extensive. The AML has an almost triangular-shaped surface and its hinge line covers approximately 1/3 of the annular circumference. The posterior leaflet has a more rectangular-shaped surface and its hinge line covers the remaining 2/3. As the AML is longer than the PML, the area of the two surfaces is equal. In the majority of normal MV, the PML is divided by two incisures into three parts, called scallops: P1 (lateral), P2 (central), P3 (medial). Conversely, the anterior leaflet has no identifiable incisures. However, surgeons call the areas of the AML opposing the corresponding scallops of the posterior leaflet A1, A2, and A3, (Figure 5A,B). Inspection of the ventricular surface of both leaflets reveals two distinct areas: the rough and clear zones. The rough zone receives the insertion of chordae tendineae and presents a “corrugate” and thicker aspect, while the clear zone, lacking chordal insertion, has a smoother, thinner and sometimes translucent appearance (Figure 5C,D). Interestingly the rough zones correspond on their atrial aspect to the area where leaflets juxtapose each other during the systole. The vast majority of chordal attachment is therefore within the coaptation area sharing the mechanical stress with the leaflets. Lack of coaptation between leaflets causes not only a mitral regurgitation but also an abnormal traction on chordae tendineae with an increasing risk of chordal rupture.

### Imaging Techniques

**Figure 6:**
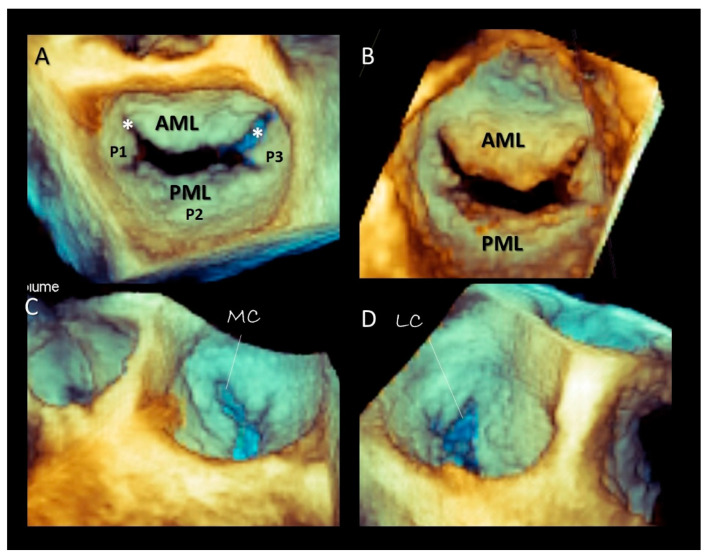
Three-dimensional TEE, the mitral valve four-basic view. (**A**) Surgical view. (**B**) View from left ventricle. (**C**) Angled lateral-to-medial view. (**D**) Angled medial-to-lateral view. AML = anterior mitral leaflet; PML = posterior mitral leaflet; MC = medial commissure (asterisk); LC = lateral commissure (asterisk).

Though in recent years CMR and CT have been increasingly used for evaluating MV apparatus, especially in the pre-operative or pre-interventional setting [19,21], there is no doubt that 2D/3D TEEs are probably the best modalities for visualizing mitral leaflets. Remarkably, once acquired, 3D volumetric data set can be rotated and angulated, allowing visualization of leaflets in a countless number of perspectives. There are at least four 3D TEE “basic” perspectives that are clinically useful: the perspective from overhead, also called “surgical view”, which provides images of leaflets equal to the surgical inspection in the operating room, the perspective from left ventricle which provides images of ventricular surface of the leaflets, the angled right-to-left and left-to-right perspectives which allow a better vision of medial and lateral commissures [22] (Figure 6). Moreover, slight angulations or rotations from these basic perspectives or selective cropping permits visualizing anatomic details of the leaflets. 

## 4. Chordal Apparatus

The chordal apparatus is characterized by a marked variability among individuals. Nevertheless, the general design is rather constant [23]. Most chordae originate from the heads of the papillary muscles, as single stems that split radially in several branches. Only the basal chordae (see below) originate directly from the wall of the left ventricle. Before inserting to the leaflets, the chordal branches form numerous interconnections, that assure a balanced distribution of forces and robust structural stability. The simplest classification divides the chordae into three groups: the first-order or marginal chordae, the second-order called strut or stay chordae, and the third-order or basal chordae. These three types of chordae exert different functions. Most of the first-order chordae insert close to the free margin of leaflets. Their rupture causes flail and significant mitral regurgitation. Interestingly, the central zone of both leaflets is a “chordal-free zone” and is the safest area for clipping percutaneously the leaflets in functional mitral regurgitation. Chordae afferent to commissures (commissural chordae) are considered first-order chordae. The second-order chordae, called also strut or stay chordae, are thicker than the marginal chordae and are attached on the border between the rough and the clear zone on both leaflets. Of particular interest are the two (or more) strut chordae inserted on the anterior leaflet at an angle of 45 degrees. Studies on normal porcine valve show that the strut chordae are under stretch either during systole or during diastole, indicating a transfer of forces from leaflets to chordae and vice versa throughout the cardiac cycle [24]. Finally, the basal chordae originate directly from the ventricular wall and insert only on the posterior leaflet. Anchoring the leaflet to the ventricular wall, these basal chordae reduce its mobility.

### Imaging Techniques

**Figure 7:**
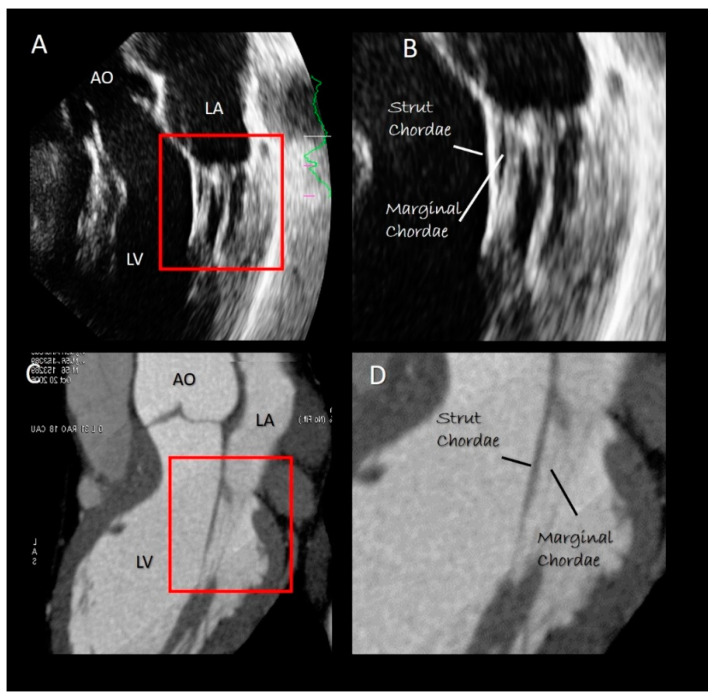
(**A**) 2D TTE and (**C**) CT cross section in long-axis view. (**B**,**D**) Magnified images of the structures inside the red rectangle of panel **A** and **C,** respectively, showing the strut and marginal chordae (see text).

Both 2D TTE/TEE echocardiography and CT may visualize the chordal apparatus. However, being cross-sectional modalities, moving chordae can be visualized only when segments of them pass through the tomographic plane (Figure 7). Theoretically, 3D TTE/TEE should be the best method for visualizing the chordal apparatus, since chordae remain within the pyramidal data set during the cardiac cycle. However, the spatial resolution of 3D TTE/TEE is poor in comparison with 2D TTE/TE. Moreover, because of the blurred artefacts, thin structures such as chordae tendineae appear thicker than they actually are [25].

## 5. Papillary Muscles

The papillary muscles (PMs) originate from the apical third of the left ventricle and are usually organized into two groups, the anterior-lateral and the posterior-medial sited below the corresponding commissures. As for the chordal apparatus, considerable variation in size, length and configuration (single PM with or without multiple heads or multiple PMs) may occur among individuals [26,27]. However, generally, the anterior-lateral is larger than the posterior-medial PM. The long axis of PMs is parallel to the long axis of left ventricle. Each of the two groups of PMs gives rise to dozens of main chordae tendineae that insert into the medial and lateral halves of both leaflets. PMs have an essential role in the closure of the mitral leaflets. When the left ventricle contracts and shortens, the simultaneous contraction of PM maintains, at constant, the distance between the tips of the PMs and the leaflets, preventing leaflet eversion during the systole. The anterior-lateral PM has a dual blood supply from both the anterior descending and the circumflex coronary arteries while the posterior-medial PM is only dependent on the coronary artery that gives origin to the posterior descending coronary. This “asymmetric” vascularization may explain different clinical scenarios. A myocardial infarction involving the posterior myocardial wall usually results in a necrosis of the posterior-medial PM (with consequent mitral regurgitation), while an anterior myocardial infarction may affect the anterior-lateral PM. Several textbooks describe PMs as arising directly from the compact myocardium layer. Recently, this belief was challenged by CT scan imaging, which revealed that PMs arise from a network of trabeculations rather than from a single pillar [28]. The attachment on a broad mesh-like architecture could protect PMs from ventricular pressure more effectively than a pillar-like attachment. Furthermore, multiple origins allow PMs to draw blood supply from numerous pathways, thus assuring a diffuse collateral perfusion. 

### Imaging Techniques

**Figure 8:**
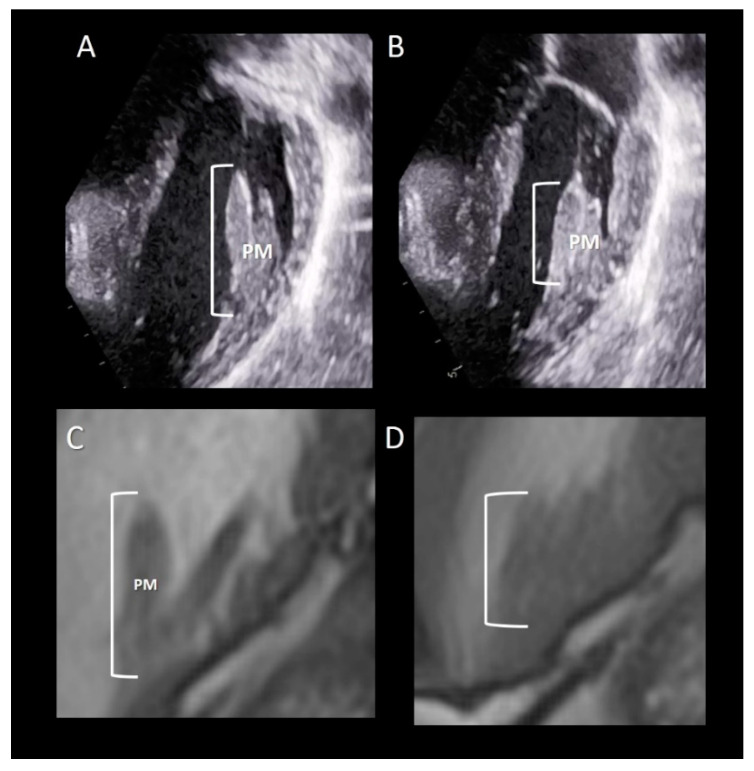
(**A**,**B**) 2D TTE and (**C**,**D**) CMR cross-sections in diastole (**A**,**C**) and in systole (**B**,**D**) showing the longitudinal contraction of papillary muscle (PM) preventing leaflet eversion during the systole (see text).

**Figure 9:**
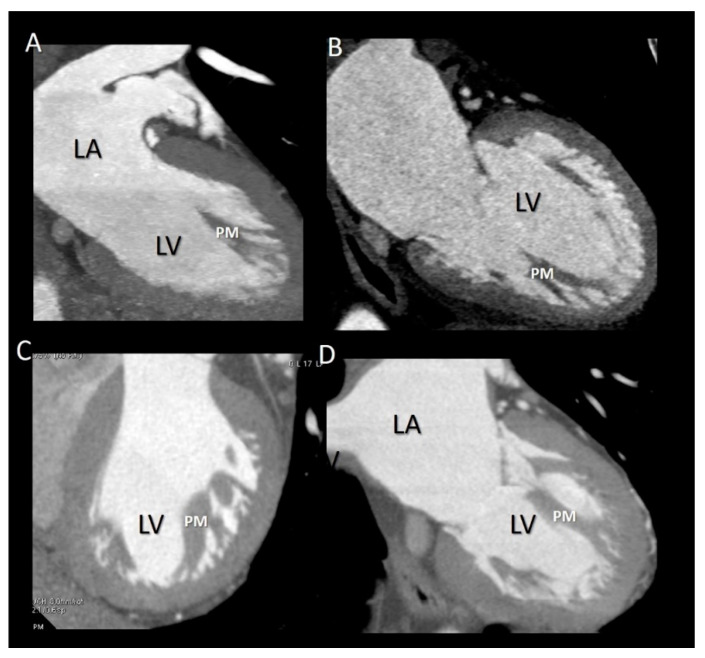
(**A**–**D**) CT representative images showing as the base of PMs do not attach to the compact myocardium but rather to a network of trabeculations.

All the three imaging modalities may beautifully illustrate PMs. Two-dimensional TTE/TEE and CMR, because of their high frame rate, may assess the shortening of the PM (Figure 8). Conversely, the high spatial resolution of CT allows for the obtaining of exceptional images of the variable anatomy of the PMs attachment on ventricular myocardium (Figure 9).

## 6. Pathological Implications

### 6.1. Mitral Annulus

#### 6.1.1. Dilation

The particular anatomical arrangement of posterior MA may explain some pathological scenarios specifically relating to this portion of the annulus. Being a “soft” structure, the posterior MA is much more exposed to annular dilatation than the more resistant anterior counterpart. As a consequence, dilation of MA does not comprise the entire annular circumference but is rather “asymmetric”, involving much more the posterior than the anterior segment. This asymmetric dilation, increasing septal–lateral diameter, impedes an effective leaflet’s coaptation with resulting mitral regurgitation. 

#### 6.1.2. Calcifications

The reason why the posterior segment of MA is affected by calcifications much more frequently and extensively than the anterior one is unclear [29]. It may be speculated that those parts of the posterior leaflet directly inserted on the ventricular myocardium may be subjected to frictions and microinjuries during the contraction and relaxation of LV. The consequent focal inflammation may extend to the adjacent AT and eventually lead to the deposition of microcalcifications. Over decades, the microscopic calcifications may coalesce into the calcific band macroscopically evident on the base of posterior leaflet replacing the AT.

#### 6.1.3. MA Disjunction

**Figure 10:**
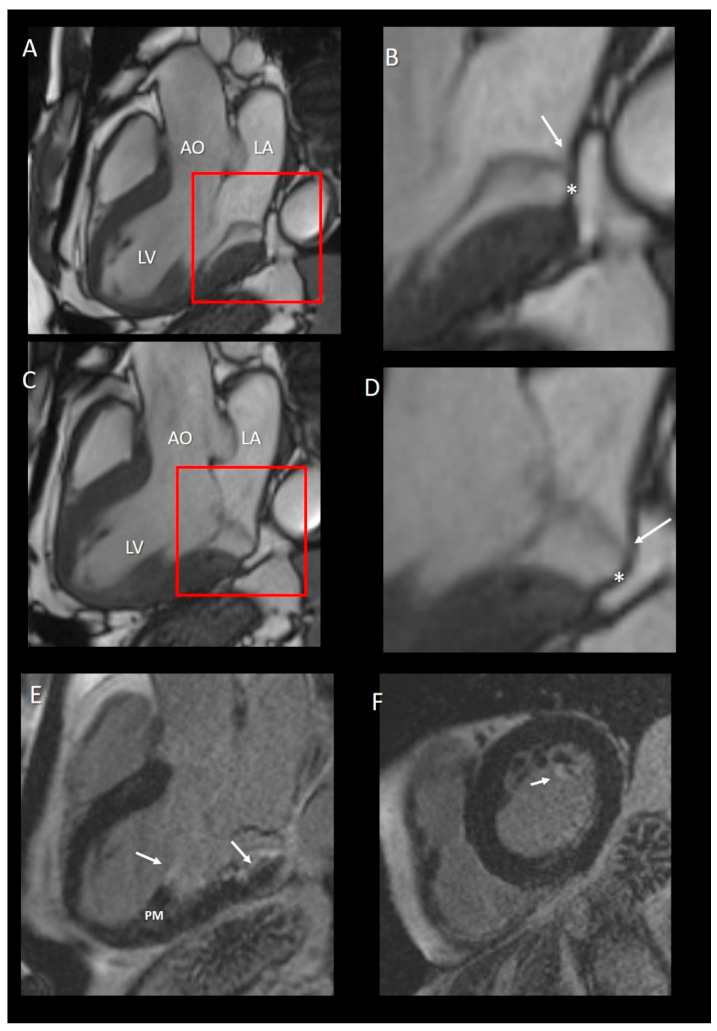
(**A**,**C**) CMR images in long-axis view in diastole (**A**) and in systole (**C**). (**B**,**D**) Magnified images of the structures in the red squares in panel A and C, respectively. The insertion of posterior leaflets on the atrial myocardium (arrow) is well visible either in diastole (panel **B**) or in systole (panel **C**). The asterisks in both **B** and **D** panel mark the curtain-like fibrous tissue. (**E**) Long-axis view late gadolinium enhancement (LGE) showing the fibrosis on the tip of papillary muscle (PM) and at the base of LV (arrows). (**F**) Short-axis view LGE showing the fibrosis on the tip of PM.

In an autoptic study based on 900 hearts, Hutchins et al. [30] found in 23 out of 25 hearts with bileaflet mitral valve prolapse (MVP), an anomalous arrangement of the hinge line of posterior leaflet: instead of being inserted on the junction of atrial and ventricular myocardium, the posterior leaflet was attached only on atrial myocardium creating an unusual “valvular-atrial junction” (Figure 10A–C). They called this arrangement mitral annulus disjunction (MAD). Interestingly, the space between the leaflet insertion and the crest of ventricular myocardium is replaced by a curtain-like fibrous tissue. Angelini et al. [12] found this atypical anatomy not only in hearts with prolapse and redundant leaflets, but also in hearts with morphological normal leaflets. MAD went unnoticed for several decades until Perazzolo Marra et al. [31] and Basso et al. [32] provided evidence that it was almost constantly present in those patients in whom MVP was associated with complex ventricular arrhythmias. This study added a new piece in the long-standing debate on arrhythmogenic MVP. Authors hypothesized that this particular arrangement would determine a myocardial stretch on those regions anatomically and functionally connected with the valve, specifically on papillary muscle (PM) tips and on basal ventricular myocardium, enhancing the propensity of MVP to cause arrhythmias. This hypothesis was confirmed by CMR and autoptic studies that demonstrated the presence of fibrous tissue either on the tip of papillary muscles or on basal ventricular myocardium. More recently, Dejgaard et al. [33] found that severe arrhythmic events, such as aborted cardiac arrest or sustained ventricular tachycardias, were related to MAD either in the presence or in the absence of MVP. Interestingly, MAD patients without MVP were more likely to have experienced severe arrhythmic events than patients with MVP. In other words, the MAD and MVP may be considered separate diseases. Further studies in larger and unselected populations are required to confirm if MAD itself is an arrhythmogenic entity.

#### 6.1.4. Endocarditis

As far as the anterior annulus is concerned, the mitral–aortic curtain is avascular and offers little resistance to bacteria, becoming a perfect “entry point” for native or prosthetic aortic leaflet endocarditis. Bacteria grow between the aortic wall layers, producing a local dissection with development of peri-annular aortic root abscesses [34] or pseudoaneurysm of the mitral–aortic intervalvular fibrosa [35]. 

### 6.2. Mitral Leaflets

In functional mitral valve regurgitation (FMR), insufficiency has been labelled as “secondary” being the result of tethered forces due to two main LV pathological scenarios, i.e., LV regional wall motion abnormalities (due to myocardial infarction) and global LV disfunction (due to idiopathic or ischemic cardiomyopathy). In idiopathic/ischemic cardiomyopathies, two factors play a significant role: the annular dilation and the reduced closing forces as opponent to tethering forces. In this setting, leaflets have been described as structurally normal and considered only a passive by-stander [36]. Though the malfunctioning LV is certainly the main cause of FMR, the belief that leaflets are structurally normal is not entirely true. In 2009, Del-Bianco et al. in an experimental study demonstrated that tethering imposed on leaflets by malfunctioning LV increases MV leaflet size and thickness of the leaflets, with cellular changes suggestive of reactivated embryonic developmental pathways [37]. Whether this mechanism is “adaptive” (i.e., the increased size improves coaptation reducing mitral regurgitation) or maladaptive [38] (i.e., the increased thickness and rigidity worsen mitral regurgitation) or even both in different moments (i.e., starting adaptive and becoming maladaptive), is still not clear. However, what is now clear is that in FMR leaflets are no longer passive by-standers but a new player that may significantly contribute to valve insufficiency. 

### 6.3. Chordae Tendineae

Rupture of first-order chordae inserted near the margins of leaflets inevitably leads to flail leaflet and severe insufficiency. This does not happen with second-order (or strut) chordae. These chordae are particularly thick and robust, but their function is not completely understood. In an animal model their transection does not result in mitral regurgitation but rather in a global LV systolic dysfunction [39]. The current hypothesis is that these chordae act maintaining a fibrous connection between the mitral valve and the papillary muscles and may contribute to the preservation of the long-axis LV shortening. In the setting of FMR, however, strut chordae are believed to exacerbate leaflet tethering. By distorting the shape of the anterior leaflet (the so-called “seagull sign”) they further exacerbate mitral regurgitation [40]. Accordingly, some authors suggest the transection of strut chordae as a complementary technique to valve annuloplasty [41,42]. 

## 7. Surgical and Interventional Implications

Annular valvuloplasty using prosthetic ring was introduced by Carpentier et al. [43] and currently prosthetic ring annuloplasty is the final act in almost all cases of mitral valve repair. Indeed, implanting a prosthetic ring around the natural annulus after leaflet’s and/or chordal repair (a) restores annular shape and size improving leaflet coaptation, (b) reduces the stress on the leaflets and on the suture line (especially once part of the leaflet has been resected), (c) maintains annular dimensions over time ensuring stability of repair and preventing recurrence of mitral regurgitation [44]. In the operating room, cardiac surgeons have a limited view of the MV. From an overhead perspective, the surgeon cannot recognize the incomplete fibrous ring described by anatomists, but rather the so-called “transition zone”, a virtual line (which corresponds to the hinge line) that separates the pale pink atrial wall, from the yellowish leaflets. The posterior fibrous string, when present, is located 2–3 mm external to this transition zone. Though not visible, this string is perceived because of its resistance to the stitches and indirectly demonstrated by its capability to hold sutures. Posteriorly, the needle should penetrate the atrial myocardium 2-mm peripheral to the transition zone, in order to preserve the motion of the leaflets, and directed towards the left ventricle to include the sparse segments of fibrous string. Anteriorly, the suture should be stitched 1 mm outside the hinge line and the stitches should be passed through the mitral–aortic curtain.

**Figure 11:**
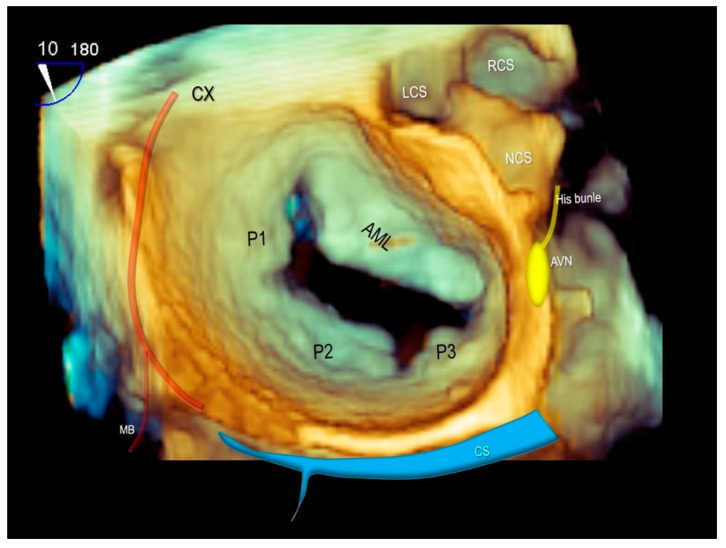
Three-dimensional TEE surgical view of mitral valve showing the anatomical relationships of mitral valve with the circumflex coronary artery (CX), the coronary sinus (CS) and the atrio-ventricular node (AVN). AML = anterior mitral leaflet, P1–P2–P3 = scallops of posterior mitral leaflet, LCS = left coronary sinus, RCS = right coronary sinus, NCS = non-coronary sinus, MB = marginal branch of CX.

Of note, both the circumflex coronary artery and the coronary sinus are closely related to the posterior mitral valve annulus and can be susceptible to perioperative injury. Furthermore, the atrio-ventricular node is above to the posteromedial commissure of the mitral valve with a potential risk of damage to the atrio-ventricular conduction (Figure 11) [45,46].

Recently, MA has been targeted for a novel device: the Cardioband™ (Edwards Lifesciences). This device, implanted percutaneously, functions as an annuloplasty band mimicking the surgical annuloplasty [47,48]. Transfemoral direct annuloplasty consists of a polyester sleeve with radiopaque markers which is fixed through multiple anchors on the “posterior annulus”. Anchors should be implanted 2–3 mm externally from the hinge line to avoid damaging posterior leaflet. As the anatomical arrangement of posterior annulus, anchors must penetrate first the atrial musculature adjacent to the hinge line, then must advance through the AT and reach the crest of ventricular myocardium where eventually they are implanted. Damage of the circumflex artery could also occur during the procedure due to cinching-related circumflex artery kinking [49].

## 8. Conclusions

This review highlights the role of non-invasive imaging techniques in illustrating the anatomy of MV apparatus. Knowledge of normal anatomical and dynamic aspects of MV is of paramount importance for a correct interpretation of the wide spectrum of patho-morphological aspects of MV diseases.
